# Advanced Synchrotron Radiation Techniques for Nanostructured Materials

**DOI:** 10.3390/nano9091279

**Published:** 2019-09-07

**Authors:** Chiara Battocchio

**Affiliations:** Department of Science, Roma Tre University, 00146 Rome, Italy; chiara.battocchio@uniroma3.it; Tel.: +39-065773-3400

Nanostructured materials exploit physical phenomena and mechanisms that cannot be derived by simply scaling down the associated bulk structures and behaviors; furthermore, new quantum effects come into play in nanosystems. The exploitation of these emerging nanoscale interactions prompts the innovative design of nanomaterials.

Understanding the behavior of materials on all length scales, from the nanostructure up to the macroscopic response, is a critical challenge for materials science. Modern analytical technologies based on the synchrotron radiation (SR) allow the non-destructive investigation of the chemical, electronic, and magnetic structure of materials in any environment. The SR facilities have developed revolutionary new ideas and experimental set-ups to characterize nanomaterials, involving spectroscopy, diffraction, scatterings, microscopy, tomography, and all kinds of highly sophisticated combinations of such investigation techniques.

This Special Issue seeks to cover all aspects of synchrotron radiation applied to the investigation of chemical, electronic, and magnetic structure of nanostructured materials. It is composed by eight research articles, that together provide not only an interesting and multi-disciplinary overview on the chemico-physical investigation of nanostructured materials carried out by state-of-the-art synchrotron radiation induced techniques, but also an exciting glance on the future perspectives of nanomaterials characterization methods. The published papers focus on the chemical, structural, and morphological characterization of nanostructured materials of different nature carried out by a wide selection of SR-induced techniques such as the X-ray photoelectron spectroscopy (XPS) [1,2] and near edge X-ray absorption fine structure (NEXAFS) [1], in situ XPS and electrochemistry [3], time-resolved X-ray scattering [4], nuclear forward scattering (NFS) of synchrotron radiation [5], soft X-ray absorption spectroscopy (sXAS) [6], X-ray fluorescence [7], X-ray diffraction [7], X-ray absorption [7] and time-resolved X-ray transmission microscopy [8].

In the following, a brief overview of the individual articles published in this Special Issue will be provided, with the aim to elicit the interest of potential readers.

In the first paper of the Special Issue [1] V. Secchi et al. exploited an SR-induced XPS and angular dependent NEXAFS to investigate the chemisorption of a self-assembling peptide (EAbuK16, i.e., H-Abu-Glu-Abu-Glu-Abu-Lys-Abu-Lys-Abu-Glu-Abu-Glu-Abu-Lys-Abu-Lys-NH_2_) onto annealed Ti25Nb10Zr alloy surfaces; the data acquired on Ti25Nb10Zr discs after incubation with self-assembling peptide solution at five different pH values allowed the investigation of the best conditions for peptide immobilization, by comparing the quality (coverage, molecular order) of the obtained nanostructured films. The X-ray photoemission spectroscopy at a high resolution, allowed by the use of SR as an X-ray source, was also exploited in the work from Y.-T. Cheng et al. [2] to investigate the embryonic stage of oxidation of an epi Ge(001)-2 × 1 by atomic oxygen and molecular O_2_. The authors observed that the topmost buckled surface with the up- and down-dimer atoms, and the first subsurface layer behaved distinctly from the bulk by exhibiting surface core-level shifts in the Ge 3d core-level spectrum, and that O_2_ molecules underwent dissociation upon reaching the epi Ge(001)-2 × 1 surface. The SR-induced XPS allowed the authors to observe that the down-dimer Ge atoms and the back-bonded subsurface atoms remained inert towards atomic O and molecular O_2_, a behavior which might account for the low reliability in the Ge-related metal-oxide-semiconductor (MOS) devices. Still using XPS, but in situ experimental conditions in the field of electrochemistry, J. Kruusma et al. [3] investigated the influence of electrode potential on the electrochemical behavior of a 1-ethyl-3-methylimidazolium tetrafluoroborate (EMImBF_4_) solution containing 5 wt% 1-ethyl-3-methylimidazolium bromide (EMImBr). The authors followed the evolution of the Br 3d_5/2_ XPS signal, collected in a 5 wt% EMImBr solution at an EMImBF_4_–vacuum interface, and were able to detect the start of the electrooxidation process of the Br^−^ anion to Br^3−^ anion and thereafter to the Br_2_ at the micro-mesoporous carbon electrode, polarized continuously at the high fixed positive potentials. Moreover, the B1s spectral region was monitored allowing to evidence B–O bond formation at E ≤ −1.17 V, parallel to the start of the electroreduction of the residual water at the micro-mesoporous carbon electrode. This study demonstrates the excellent potentiality of in situ SR-induced spectroscopies in investigating the details of electrochemical processes in operando. In situ observations of chemico-physical phenomena are nowadays allowed by several techniques; in this context, D. Smrčka et al. [5] report about the application of nuclear forward scattering (NFS) of synchrotron radiation to the in situ study of crystallization of metallic glasses. By performing in situ temperature experiments in the presence and absence of an applied magnetic field, they are able to carry on the investigation not only from the structural point of view, evidencing the formation of nanocrystalline grains, but also to observe the evolution of the corresponding hyperfine interactions, and the differences in the NFS spectrum evolution induced by the experimental conditions (i.e., temperature and magnetic field). Temperature-related effects on the nanostructured materials behavior are also studied by A. Plech et al. [4], which propose an innovative method to investigate the thermal conductivity of thin films exploiting the high time-resolution in scattering analysis. In their manuscript, the authors apply transient pump-probe detection of dissipation of laser-induced heating (TDXTS) to investigate two extreme examples of phononic barriers, isotopically modulated silicon multilayers with very small acoustic impedance mismatch and silicon-molybdenum multilayers, which show a high resistivity; the reliable results obtained and described allow the validation of the proposed method. In [6], X. Long et al. focus their attention on the structural investigation of nanocrystalline multivalent metal spinels carried out by sXAS; the object of this research is to identify the active sites in cubic and tetragonal Co_x_Mn_3-x_O_4_ (x = 1, 1.5, 2) spinel oxides (a family of highly active catalysts for the oxygen reduction reaction—ORR) and to understand their reaction mechanisms, since these aspects are essential to explore novel transition metal oxides catalysts and further promote their catalytic efficiency. The authors demonstrate that the ORR activity for oxide catalysts primarily correlates to the partial covalency between the O 2p orbital with Mn^4+^ 3d t_2g_-down/e_g_-up, Mn^3+^ 3d e_g_-up and Co^3+^ 3d e_g_-up orbitals in octahedron, which can be directly revealed by the O K-edge sXAS. The findings reported in this publication highlight the importance of electronic structure in controlling the oxide catalytic activity. The X-ray absorption apectroscopy is also used by E. Secco et al. [7] to study individual nanowires (NWs) containing non-polar GaN/InGaN multi-quantum-wells, in a multi-technique investigation carried out by hard X-ray spectroscopies: X-ray fluorescence, X-ray diffraction, and X-ray absorption. Thanks to the improvements in the spatial resolution of synchrotron-based X-ray probes, that have now reached the nano-scale, the authors were able to probe the chemical composition of the nanowires as well as to describe the nanomaterial structure observing that while the GaN core and barriers appear fully relaxed, there is an induced strain in InGaN layers corresponding to a perfect lattice matching with the GaN core. Such strain, together with the observed inhomogeneous alloy distribution, affects the photoluminescence spectrum of non-polar InGaN quantum wells but still exhibits a reasonable 20% relative internal quantum efficiency. This study evidences how a multi-technique approach, allowed by the synchrotron radiation facilities, allows for a wide and accurate description of highly complicated materials, such as the ones proposed by the authors. The most recent publication in the Special Issue is the paper from T. Schaffers et al. [8] describing the application of a time-resolved detection scheme in the scanning transmission X-ray microscopy (STXM) to measure the element resolved ferromagnetic resonance (FMR) at microwave frequencies up to 10 GHz and a spatial resolution down to 20 nm at two different synchrotrons. The authors discuss different methods to separate the contribution of the background from the dynamic magnetic contrast based on the X-ray magnetic circular dichroism (XMCD) effect, and describe how the relative phase between the GHz microwave excitation and the X-ray pulses generated by the synchrotron, as well as the opening angle of the precession at FMR, can be quantified. In conclusion, the authors demonstrated how the dynamic magnetic contrast in time-resolved STXM has the potential to be a powerful tool to study the linear and nonlinear, magnetic excitations in magnetic micro- and nano-structures with unique spatial-temporal resolution in combination with element selectivity.

To conclude this overview on the papers published in the Special Issue “Advanced Synchrotron Radiation Techniques for Nanostructured Materials”, I am confident that the readers will enjoy these contributions and may be able to find inspiration for their research within this Special Issue.
